# *APOE* Polymorphisms Contribute to Reduced Atorvastatin Response in Chilean Amerindian Subjects

**DOI:** 10.3390/ijms16047890

**Published:** 2015-04-09

**Authors:** Jenny Lagos, Tomás Zambrano, Alexy Rosales, Luis A. Salazar

**Affiliations:** Center of Molecular Biology and Pharmacogenetics, Department of Basic Sciences, Scientific and Technological Bioresource Nucleus, Universidad de La Frontera (BIOREN-UFRO), Av. Francisco Salazar 01145, 4811230 Temuco, Chile; E-Mails: tm.jenny.lagos@gmail.com (J.L.); t.andreszambrano@gmail.com (T.Z.); tm.arosales@gmail.com (A.R.)

**Keywords:** atorvastatin, *APOE*, *LDLR*, polymorphisms, pharmacogenetics, Amerindian

## Abstract

Genetic factors can determine the high variability observed in response to lipid-lowering therapy with statins. Nonetheless, the frequency of single nucleotide polymorphisms (SNPs) and their impact can vary due to ethnicity. Because the Chilean population carries a strong Amerindian background, the objective of this study was to evaluate the influence of apolipoprotein E (*APOE*) variants (rs429358, rs7412) and the 1959C>T SNP (rs5925) in the low-density lipoprotein receptor (*LDLR*) in response to atorvastatin treatment in hypercholesterolemic individuals. A hundred and thirty nine subjects undergoing statin therapy were included. Identification of Amerindian mtDNA haplogroups was determined by polymerase chain reaction (PCR) and PCR followed by restriction fragment length polymorphism (RFLP), respectively. SNPs were determined by PCR-RFLP. Out of the 139 individuals studied, 84.4% had an Amerindian background, according to mtDNA analysis. In relation to *APOE* variants, carriers of the E3/4 genotype presented lower cholesterol reduction compared to genotype E3/3 (LDL-C: −18% *vs*. −29%, *p* ˂ 0.001). On the other hand, the *LDLR* rs5925 SNP was not related to atorvastatin response (*p* = 0.5760). Our results suggest that *APOE* SNPs are potential predictors to atorvastatin therapy in Amerindian Chilean subjects.

## 1. Introduction

Elevated plasma levels of low-density lipoprotein cholesterol (LDL-C) are strongly associated as a key risk factor for cardiovascular disease. Clinical management of hypercholesterolemia using statins not only improves the lipid profile, but also has multiple atheroprotective effects, reducing mortality and the incidence of cardiovascular events [1,2].

The effectiveness of statin therapy has been underlined by several studies. However, when an identical dose is used, a spectrum of different responses among treated individuals emerges. This interindividual variability in therapeutic response may be determined by several factors, such as biological and physiological conditions of the patient, treatment adherence, age, gender, ethnicity and genetic factors specific to each individual [3,4,5]. In this sense, pharmacogenetic studies have established that lipid-lowering response to statins can be affected by the presence of genetic variations in genes involved in central cholesterol-related processes like intestinal absorption, metabolism and in genes regulating the pharmacokinetics and pharmacodynamics of statins [6,7,8,9,10]. However, results obtained are not always reproducible, which could be partially influenced by the ethnical characteristics of the population studied [11]. Moreover, most pharmacogenetic investigations have been performed in Caucasian, Asian and African populations [12,13]. In contrast, Chilean subjects have an ethnic background differing from other world populations, reporting a high percentage of Amerindian population [14,15]. Characterization of this ethnical group can be achieved by means of mitochondrial DNA markers (mtDNA), making possible the identification of four haplogroups called A, B, C and D, through recognition of genetic variants [16,17].

As atorvastatin is the leading statin used in Chilean healthcare services, searching for pharmacogenetic markers of atorvastatin response to the characteristic Chilean population is of great interest. So, this work evaluated the influence of apolipoprotein E (*APOE*) genotypes on the response to atorvastatin. In addition, as the low-density lipoprotein receptor (*LDLR*) is another locus robustly associated to variability in LDL-C and statin response, the polymorphism rs5925, previously associated with low TC, LDL-C, HDL-C and TG in different groups of individuals [18,19], was also assessed in this study.

## 2. Results

Clinical and demographic characteristics of the study group are summarized in Table 1. The reduction of plasma values of TC (274 ± 18.3 *vs.* 224 ± 26.0 mg/dL; −18.2%), LDL-C (185 ± 17.5 *vs*. 137 ± 26.1 mg/dL; −25.9%) and TG (213 ± 50.5 *vs*. 166 ± 48.4 mg/dL; −22.0%) were significant after completion of the treatment (*p* < 0.001). In addition, we observed an increase in HDL-C levels after atorvastatin therapy (46.4 ± 8.8 *vs.* 54.1 ± 6.7 mg/dL; +14.8%). According to the ethnic characterization achieved by mitochondrial DNA analysis, we observed that 116 individuals (84.4%) of the studied population was Amerindian (Table 1). Those individuals who did not have any of the A, B, C and D haplogroups were classified as non-Amerindian (15.6%). Due to the low frequency of non-Amerindian (*n* = 16), it was not possible to perform a comparative analysis of statin response between Amerindian and non-Amerindian subjects.

Genotype distribution and relative allele frequencies for the studied polymorphisms are shown in Table 2. There were no differences in cholesterol levels when the *LDLR* rs5925 variant was present (Table 3). However, carriers of E3/4 genotype of *APOE* had a lower TC, LDL-C and TG reduction when compared to the E3/3 genotype (Table 4). Genotypes E2/3 and E2/4 were not included in the comparative analysis due to their low frequency (2.5% and 2%, respectively).

**Table 1:** Clinical and demographic characteristics of the study group.

Parameter	*n* = 139
Age (years)	56.4 ± 10.7
Men/Women (*n*)	87/52
BMI (kg/m^2^)	25.6 ± 2.7
Systolic blood pressure (mmHg)	106.8 ± 12.3
Diastolic blood pressure (mmHg)	72.7 ± 9.2
TC (mg/dL)	274 ± 18.3
TG (mg/dL)	213 ± 50.5
LDL-C (mg/dL)	185 ± 17.5
HDL-C (mg/dL)	46 ± 8.8
Amerindian mtDNA haplogroups (%)	
Haplogroup A	1.4
Haplogroup B	29.0
Haplogroup C	32.0
Haplogroup D	22.0
Non-Amerindians	15.6

TC: total cholesterol; LDL-C: low-density lipoprotein cholesterol; HDL-C: high-density lipoprotein cholesterol; TG: triglycerides.

**Table 2:** Genotype distribution and relative allele frequencies for the polymorphisms studied in Amerindian hypercholesterolemic subjects (*n* = 116).

	Genotypes						Allele Frequency			
***LDLR***	CC		CT		TT		C		T	
*(rs5925)*	22.4%		66.4%		11.2%		0.556		0.444	
***APOE***	2/3	3/3		3/4		2/4	ε2	ε3		ε4
(rs429358, rs7412)	2.5%	61.5%		34%		2%	0.021	0.802		0.177

Genotypes E2/2 and E4/4 were not found.

**Table 3:** Lipid profile and response to atorvastatin treatment (10 mg/day/4weeks) according to the low-density lipoprotein receptor (*LDLR*) rs5925 polymorphism in hypercholesterolemic Amerindian subjects.

Lipids (mg/dL)	Condition		Genotypes		*p* -Value
		CC (*n* = 25)	CT (*n* = 78)	TT (*n* = 13)	
TC	*basal*	271 ± 14.4	273 ± 18.2	283 ± 22.1	0.1080
	*treatment*	211 ± 26.2	226 ± 27.3	224 ± 26.5	0.2423
	*% change*	−22.0 ± 10.7	−17 ± 10.6	−20.0 ± 10.8	0.3054
LDL-C	*basal*	190 ± 16.1	183 ± 17.8	192 ± 20.0	0.1989
	*treatment*	127 ± 28.6	139 ± 27.5	137 ± 27.0	0.4655
	*% change*	−32.0 ± 18.0	−24 ± 15.9	−28.0 ± 15.1	0.2258
HDL-C	*basal*	48 ± 6.0	47 ± 9.4	46 ± 7.8	0.2643
	*treatment*	55 ± 6.0	54 ± 7.0	53 ± 6.3	0.7850
	*% change*	15.0 ± 16.2	18.0 ± 14.7	18.0 ± 17.1	0.9576
TG	*basal*	197 ± 53	217 ± 48.7	224 ± 51.0	0.1047
	*treatment*	146 ± 48.6	167 ± 49.5	172 ± 48.9	0.2499
	*% change*	−25.0 ± 24.6	−23.0 ± 17.9	−22.0 ± 22.9	0.2305

Values are expressed as mean ± standard deviation; TC: total cholesterol; LDL-C: low-density lipoprotein cholesterol; HDL-C: high-density lipoprotein cholesterol; TG: triglycerides; *p*-values from ANOVA.

**Table 4:** Lipid profile and response to atorvastatin treatment (10 mg/day/4 weeks) according to apolipoprotein E (*APOE*) Genotypes in hypercholesterolemic Amerindian subjects.

Lipids (mg/dL)	Condition	Genotypes		*p*-Value
		E3/3 (*n* = 72)	E3/4 (*n* = 40)	
TC	*basal*	276 ± 19.2	275 ± 18.7	0.2630
	*treatment*	218 ± 23.3	240 ± 26.8	0.0004
	*% change*	−21.0 ± 8.9	−12.0 ± 10.9	0.0008
LDL-C	*basal*	188 ± 18.1	183 ± 18.4	0.4561
	*treatment*	132 ± 25.3	148 ± 25.8	0.0030
	*% change*	−29.0 ± 14.4	−18.0 ± 15.9	0.0046
HDL-C	*basal*	48 ± 7.8	51 ± 11.1	0.1626
	*treatment*	54 ± 6.8	56 ± 7.5	0.1352
	*% change*	20.0 ± 14.8	12.0 ± 15.2	0.0020
TG	*basal*	215 ± 50.2	209 ± 53.9	0.5598
	*treatment*	160 ± 51.4	181 ± 45.8	0.0263
	*% change*	−25.0 ± 20.0	−12.0 ± 18.1	0.0127

Values are expressed as mean ± standard deviation; TC: total cholesterol; LDL-C: low-density lipoprotein cholesterol; HDL-C: high-density lipoprotein cholesterol; TG: triglycerides; *p*-values from paired *t*-test. Genotypes 2/3 and 2/4 were not subjected to statistical analysis due to their low frequency (2.5% and 2.0%, respectively).

## 3. Discussion

Atorvastatin is effective in controlling high cholesterol and reducing cardiovascular risk. The overall LDL-C reduction can reach up to 37% in subjects using doses of 10 mg/day for 4 weeks, regardless of cholesterol levels before starting the therapy. However, the response is variable between individuals, possibly due to factors such as gender, ethnicity or genetic composition [11]. As *LDLR* and *APOE* proteins hold important roles in lipid metabolism and the mechanism of action of statins, we analyzed the influence of the *LDLR* rs5925 SNP and *APOE* genotypes on atorvastatin response in Chilean population with Amerindian background.

The main goal of statin therapy is the competitive and selective inhibition of the 3-hydroxy-3-methylglutaryl-Coenzyme A reductase (HMG-CoA reductase) enzyme. This step determines the blocking of intracellular lipid synthesis and promotes the activation of a transcription factor known as Sterol Regulatory Element-Binding Proteins (SREBPs), inducing the expression of *LDLR* [20]. Consequently, *LDLR* activity increase the number of receptors bound to the cell membrane that interacts with LDL, enhancing the clearance of this lipid fraction [20]. A major factor in this interaction is the presence of *APOE*, which depending on its isoform, influences the binding affinity between the ligand and its receptor. APOE is a structural protein of lipoproteins, mediating the binding with the *LDLR* [21]. Several studies have replicated the association between the C allele of the rs5925 SNP and higher levels of cholesterol [22,23]. This same finding was reported recently by Long *et al.* [19], demonstrating that the CC genotype associates to a higher concentration of total and LDL-C when compared to CT and TT genotypes. In Brazil, it was demonstrated that among patients treated with fluvastatin, C allele carriers had higher lipid levels and lower cholesterol reduction [6]. However, our investigation did not show this association in Chilean Amerindian subjects. The different ethnic background in Chileans may be an interesting precedent that could condition the lack of association to treatment response and to hypercholesterolemia. Importantly, this finding is the first study of *LDLR* SNP in Chileans associated to the pharmacogenetics of statins. As this variant did not show a relation with response, more studies exploring genetic variations in this receptor are needed to extend the current knowledge on its function and relationship to the therapeutic efficacy of atorvastatin.

In relation to the pharmacogenetics of statin therapy, one of the variants demonstrating more reproducibility with treatment response is the genotype E3/4 of *APOE*. This apolipoprotein is polymorphic, presenting three different isoforms (E2, E3 and E4) that are determined by six possible genotypes (E2/2, E2/3, E2/4, E3/3, E3/4, E4/4) from 3 different alleles (e2, e3 and e4). The three alleles are the result of nucleotide substitutions in codons 112 and 158 of exon 4 of the *APOE* gene [21]. While the most common isoform of apolipoprotein E is E3, current evidence shows that the E2 isoform would be associated with higher levels of LDL-C reduction compared to E3 and E4. Also, the E4 isoform has been associated with lower response to treatment [24,25]. Similarly, we also observed a lower lipid-lowering effect for the E3/4 genotype in response to atorvastatin, correlating this finding to a previous report documented by Baptista *et al.* [26]. The different *APOE* genotypes can determine various degrees of receptor affinity [21,24]. This finding is interesting in relation to the effectiveness of statins, since this drug promotes increased synthesis of receptors, and in the case of *APOE* E4, effectiveness would be limited by failure of union between ligand and receptor, independent of the number of receptors.

In addition to the improved lipid profile following atorvastatin therapy, decreasing plasma TC, LDL-C and TG, we also observed a significant influence of *APOE* E3/4 genotype on TC and TG reduction. Several epidemiological studies have established a positive relationship between TG levels and CHD. Furthermore, two meta-analyses indicate that elevated TG are an independent risk factor for the development of coronary heart disease (CHD) [27,28], establishing an atherogenic role for triglyceride-rich lipoproteins and thus, conferring increasing interest to the clinical significance of *APOE* variants and the enhanced lipid reduction identified in our work.

Identification of mitochondrial DNA haplogroups determined that 84.4% of the subjects had an Amerindian background. This finding is consistent with a prior result, where an 85% of Amerindian individuals from Southern Chile was ascertain to have this ethnicity [29]. Currently, there are associations between polymorphisms in the mitochondrial genome and increased levels of LDL-C [26,30]. Nevertheless, in a previous report, we did not find an association of Amerindian mtDNA haplogroups with atorvastatin response [29].

A concern that could be limiting the results obtained is the reduced number of individuals included, which could be providing some bias. Therefore, additional studies assessing a greater cohort are key in order to corroborate and reproduce consistently the influence of *APOE* genotypes on plasma lipid levels. However, our findings highlight the identification of the E3/4 genotype as a potential and useful biomarker of atorvastatin response. This genotype is clinically informative; allowing the use of different treatment strategies, such as the use of higher doses of atorvastatin, complementation with an inhibitor of intestinal cholesterol absorption or simply receives different pharmacologic therapy to achieve the expected therapeutic goals in hypercholesterolemic patients.

In summary, our results partially explain the variability of therapeutic response to atorvastatin therapy in a Chilean cohort, demonstrating that the presence of genotype E3/4 of *APOE* determines a lower response to treatment with atorvastatin in Amerindian subjects. Taking into account that an adequate control of dyslipidemia contributes to improving mortality, morbidity and associated cardiovascular disease, it becomes evident the need of pharmacogenetic studies to elucidate other determinants of response to statin therapy in Chilean population.

## 4. Experimental Section

### 4.1. Subjects

As statins are the main lipid-lowering medication currently prescribed, being also safe and well tolerated, hypercholesterolemic patients (*n* = 139), diagnosed according to the NCEP criteria [31] were selected from the Federico Thieme Health Center (Región de La Araucanía, Chile) and treated with 10 mg/day of atorvastatin during 4 weeks. None of the subjects had diabetes, hepatic, kidney, endocrinological or malignant disease, nor was receiving concomitant lipid-lowering therapy. Patients using medication that could affect the lipid profile, such as beta-blockers and diuretics, were excluded. All participants voluntarily signed an informed consent and the local Scientific Ethics Committee approved the study protocol.

### 4.2. Biochemical Determinations

Blood samples were obtained by venipuncture following a 12-h overnight fast. Biochemical measurements were determined by standard enzymatic-colorimetric methods (Human, Germany) and low-density lipoprotein cholesterol was calculated using Friedewald’s formula, when triglycerides did not exceed 400 mg/dL (4.8 mmol/L). The accuracy of biochemical determinations was controlled using normal and pathological commercial serums (Human, Germany).

### 4.3. Molecular Analysis

Genomic DNA was extracted from blood leukocytes by a salting out procedure optimized by Salazar *et al*. [32]. Genotyping was performed by PCR-RFLP.

### 4.4. mtDNA Haplogroups Genotyping

The procedure was completed according to Moraga *et al*. [14]. Briefly, to determine the presence or absence of the three characteristic polymorphic restriction sites of haplogroups A, C and D of Amerindian population, we used 3 specific endonucleases (*Hae*III, *Hinc*II *and Alu*I). Finally, restriction fragments were evaluated in a 3% agarose gel electrophoresis followed by ethidium bromide staining. In order to define the haplogroup B, a deletion of one out of two 9 bp repeats located in the intergenic region V was visualized in a 10% polyacrylamide gel electrophoresis directly from the PCR products obtained.

### 4.5. LDLR rs5925 Genotyping

Genotyping was carried out through PCR amplification using specific primers (Forward: 5'-GTCATCTTCCTTGCCTGTTTAG-3'; Reverse: 5'-TTTCCACAAGGAGTTTTCAAGTT-3'), allowing amplification of a 228 bp within the sequence of the 13th exon. PCR amplification reactions were performed in a final volume of 25 µL containing 50 ng of genomic DNA, 200 nM of each primer, 0.2 mM of dNTPs (dATP, dTTP, dGTP, dCTP), 1 unit of *Taq* DNA polymerase, 2 mM of MgCl_2_, PCR buffer (KCl 50 mM, 2 mM MgCl_2_, Tris-HCl, pH 9.0) and sterile deionized water. The method was carried out in a Mycycler TM thermocycler (BioRad Laboratories Inc., Hercules, CA, USA) under the following temperatures scheme: initial denaturation at 98 °C for 3 min followed by 35 cycles consisting on 95 °C for 1 min (denaturation), 66 °C for 1 min (annealing) and 72 °C for 1 min (extension) with a final extension at 72 °C for 10 min. Amplification products were evaluated in a 2% agarose gel electrophoresis stained with ethidium bromide and visualized in an UV transilluminator. PCR products were submitted to enzymatic digestion with *Eco*47I (Fermentas, Lituania) in the following reaction conditions for a final volume of 15 µL: 1 unit of enzyme (0.1 µL), 1.5 µL of enzyme buffer, 7 µL of PCR product and sterile deionized water. Reactions were incubated at 37 °C for 12 h Digestion products were revealed by 2% agarose gel electrophoresis, subsequently stained with ethidium bromide and visualized using an UV transilluminator.

### 4.6. APOE Isoforms Genotyping

PCR amplification assays using specific primers were performed (Forward: 5'-CTGACCCCGGTGGCGGA-3', Reverse: 5'-GGGGATGGGGCTGAGGC-3'), amplificating a 291 bp sequence within exon 4 of the gene. PCR amplification reactions were performed in a final volume of 25 µL containing 50 ng of genomic DNA, 200 nM of each primer, 0.2 mM of dNTPs (dATP, dTTP, dGTP, dCTP), 1 unit of *Taq* DNA polymerase, 1.5 mM of MgCl_2_, PCR buffer (KCl 50 mM, 2 mM MgCl_2_, Tris-HCl, pH 9.0), 0.75 µL of DMSO and sterile deionized water. Amplification was carried out in a Mycycler TM thermocycler (BioRad Laboratories Inc.) under the following temperatures scheme: initial denaturation at 95 °C for 5 min, 30 cycles: 95 °C for 1 min (denaturation), 62 °C for 1 min (annealing), 72 °C for 1 min (extension) and a final extension at 72 °C for 5 min Amplification products were evaluated in a 2% agarose gel electrophoresis stained with ethidium bromide and visualized in a UV transilluminator. PCR products were submitted to enzymatic digestion with *Hha*I (Fermentas, Lituania) in the following reaction conditions for a final volume of 15:1 unit of enzyme (0.1 µL), 1.5 µL of enzyme buffer, 7 µL of PCR product and sterile deionized water. Reactions were incubated at 37 °C for 12 h. Following this, digestion products were revealed by 10% polyacrylamide gel electrophoresis, and were subsequently stained with ethidium bromide and visualized using an UV transilluminator.

### 4.7. Data Analysis

Results were analyzed using the Sigma Stat statistical software v. 2.0 (Jandel Sci, San Rafael, CA, USA). All variables were analyzed descriptively. Comparison of continuous variables was performed using Student’s *t* test or ANOVA one-way test. Comparison between proportions was evaluated by Chi-square or Fisher’s exact test. Genotype and allele frequencies were obtained by direct gene counting. The statistical significance considered was α = 0.05.
